# Relationship Between Nutritional Parameters and Quality of Life in Patients With End-Stage Kidney Disease on In-Centre Haemodialysis in Al-Ahsa, Saudi Arabia

**DOI:** 10.7759/cureus.32146

**Published:** 2022-12-02

**Authors:** Mohammed Y Almulhim, Ali A Alkuwaiti, Abdullah Y Alhabrati, Ridha K Alomran, Ali M Alaithan, Hassan A Alsahaf, Muthana Alsahlawi, Sayed I Ali

**Affiliations:** 1 Internal Medicine, Faculty of Medicine, King Faisal University, Al-Ahsa, SAU; 2 Medicine, King Faisal University, Al-Ahsa, SAU; 3 Internal Medicine, Johns Hopkins Aramco Healthcare (JHAH), Dhahran, SAU; 4 Family and Community Medicine, Faculty of Biostatistics, King Faisal University, Al-Ahsa, SAU

**Keywords:** al-ahsa, saudi arabia, haemodialysis (hd), end-stage kidney disease, quality of life (qol)

## Abstract

Introduction: Nutrition threatens the quality of life (QOL) in end-stage kidney disease (ESKD) patients on haemodialysis (HD). The aim of the study is to assess which nutritional parameters associate with the health-related QOL category score using Arabic translated, adapted, and validated version of the Kidney Disease Quality of Life Short-Form 36 (KDQOL-36).

Methods: We carried out a cross-sectional study with a total of 60 ESKD patients in one haemodialysis centre in Al-Ahsa, Saudi Arabia. The health-related QOL of the five components of the KDQOL-36 are as follows: physical component score (PCS), mental component score (MCS), the burden of kidney disease (BKD), symptoms and problems of kidney disease (SPKD), and effects kidney disease (EKD) which were grouped into three categories (good, average and poor) based on the score.

Results: Of the 60 HD patients, 43 (71.7%) were between 19 and 59 years old and 41 patients (68.3%) were males. This study revealed that high haemoglobin impact PCS, MCS, SPKD, and EKD in favour of poor health-related QOL, but normal haemoglobin impacts BKD in favour of good QOL. In addition, patients who had a normal serum albumin level had good scores for MCS, BKD, SPKD, and EKD. Also, there was a significant association between Kt/V value with EKD (p=0.001).

Conclusion: According to the findings of this study, patients with HD have poor health-related QOL outcomes than those with good nutritional status. This finding demonstrates the critical need for further articles to study this particular group of population.

## Introduction

Chronic kidney disease (CKD) impacts 8%-16% of individuals globally [1,2]. CKD is characterized by the presence of structural or functional kidney defects such as GFR lower than 60 mL/min/1.73 m^2 ^- that has continued for more than three months, albuminuria of 30 mg per day, or indicators of kidney impairment such as haematuria lasting for more than three months [3]. Internationally, CKD is mainly caused by diabetes and/or hypertension, but there are other factors that can cause CKD such as glomerulonephritis, infection, and environmental exposures that are widespread in developing countries such as air pollution and herbal remedies [2]. Diagnosing and treating these patients is critical since advanced CKD can result in serious complications including cardiovascular disease, end-stage kidney disease (ESKD), and death [4-6]. ESKD is most commonly treated by conventional haemodialysis (HD) [7]. In the Middle East, the overall combined prevalence of ESKD was calculated as 360 per million individuals. In Saudi Arabia, the prevalence is 540 per million individuals, of which 92.8% are on HD therapy [8].

HD patients with malnutrition have a significant decrease in the levels of serum albumin, phosphate, haemoglobin, and residual renal function [9]. The albumin blood level is a biomarker widely used as nutritional status in CKD patients although it is influenced by other conditions like fluid status, infection, inflammation, and proteinuria [10]. The malnutrition incidence is between 18% and 75% in HD patients [11]. Malnutrition is 2.6 times more likely in females than males, and haemodialysis patients who have been on dialysis for more than five years have a 12.5-fold increased risk of malnourishment [12]. The prolonged duration of haemodialysis is associated with a significantly lower nutritional state [9]. Malnutrition is also one of the factors that threaten health-related QOL since it is associated with a higher rate of hospitalizations [11,12]. Many factors may participate in the protein-energy wasting syndrome in patients with kidney disease, including the presence of inflammation, academia, inadequate diet, and oxidative stress [13,14]. A study conducted in the Gulf Cooperation Council (GCC) countries showed that a low albumin level of <3.2 g/dL was correlated with lower QOL and a high mortality rate [15]. Another study showed that ESKD patients with hypoalbuminemia and comorbidity had a lower score on the KDQOL questionnaire [16]. Also, blood cell count is an important parameter in haemodialysis patients, especially haemoglobin levels. The risk of mortality increased when the haemoglobin level was lower than 10 g/dL [17]. The concept of a health-related QOL contains numerous aspects that include areas linked to social well-being, physical, and psychological aspects that are the subjective and primary focus for assessing the impact of health status on health-related QOL [18,19]. Studies using the KDQOL tool questionnaire or similar tools have shown that those who are treated with dialysis have a poor score of health-related QOL in comparison to the population [20-22] In addition, health-related QOL can serve as a prognostic measure and predictor for survival among dialysis patients as a study showed that a lower health-related QOL is associated with higher hospitalization rates and worse survival [20,23]. No previous assessment of QOL in Al-Ahsa, Saudi Arabia, HD patients has been performed. The KDQOL-36 is composed of 36 questions divided into four parts [24]. The first part (items 1 - 12) comprises questions about activity, anxiety, ability to accomplish tasks, depression, energy, general health, and social activities that comprise the physical component score (PCS) and mental component score (MCS) scales. The second part (items 13 - 16) includes questions about what takes up time, causes frustration, makes the participants feel like a burden, and how kidney disease interferes with daily life activity that comprises the burden of kidney disease (BKD) scale. The third part (items 17 - 28) includes questions about how bothered the participants feel by muscles or chest pain, dry skin, problems with dialysis access burden, lack of appetite, shortness of breath, faintness, itchy skin, feeling washed out or drained, numbness over the body, or nausea, which comprises the symptoms and problems of kidney disease (SPKD) scale. The last part (items 29 - 36) includes questions about how bothered the participants feel by feeling dependent on the medical staff, dietary and fluid restrictions, ability to work around the house or travel, sexual life, worries, and personal appearance, which comprises effects kidney disease (EKD) scale [24].

The primary aim of this study is to assess the nutritional parameters and the levels of the QOL and determine their association in patients with ESKD using Arabic translated, adapted, and validated version of the Kidney Disease Quality of Life Short-Form 36 (KDQOL-SF-36).

## Materials and methods

Study duration, setting, and sampling

This study was conducted between June 7 and June 19, 2021 at the Al Jaber Kidney Center in Al-Ahsa, Saudi Arabia, on patients who accepted to participate in the study, were in-centre the HD and met our inclusion and exclusion requirements. Our area of research is appropriate for investigating such emerging public health problems because the eastern region of Saudi Arabia has a growing population, which assists policymakers by exploring scientific knowledge. The patients were chosen using a convenient non-randomized sampling technique and were adult patients treated with HD who are attending in-centre HD. This study eventually included 60 samples.

Study subject and design

This observational study was conducted among 60 HD patients to evaluate and investigate the nutritional parameters with the levels of the heath-related QOL and determine their association with ESKD patients. The patients were enrolled after consenting by completing the Arabic version of the KDQOL-36 questionnaire and meeting the following inclusion criteria: (i) On in-centre HD, (ii) Saudi citizens from Al-Ahsa by birth, (iii) adults (18 years old and older) and (IV) on HD for more than one year. Patients who refused to participate in this study or who had psychiatric issues or serious medical conditions were not allowed to take part in the study.

Content of the study tool

The survey tool used in this study consisted of three parts: (i) patients’ demographic characteristics (ii) assessment of patients’ QOL using Arabic translated, adapted, and validated version of the KDQOL-SF-36 questionnaire with 36 items and (iii) nutritional parameters including haemoglobin and albumin. The first section of the questionnaire consists of patients’ demographic characteristics including gender and age group. The second section is the patients' QOL was assessed with the KDQOL-SF-36 questionnaire. The KDQOL-36 is a validated and widely used questionnaire with five components: the PCS, MCS, BKD, SPKD, and effects of kidney disease (EKD). A total of 36 questions. The endpoints were scored and categorized as poor, average, or good. Each question is coded and then score on a scale of 0-100; higher scores indicate better health-related QOL. Additional collected parameters were the duration of HD, the number of sessions per week, the duration of the sessions, residual kidney function, the type of dialysis access, Kt/v, and chronic diseases. The third part is nutritional parameters which were data collected the prior six months from the HD centre patients’ electronic medical records.

Questionnaire validity

The study's authors examined all earlier studies on patients who went through HD and impairment of nutritional parameters. The authors searched different databases including Cochrane Library, PubMed, WHO, and Google Scholar until November 2, 2022. The questionnaire was built using the most relevant articles. The internal consistency of the translated version of the KDQOL-36 questionnaire was assessed using Cronbach's alpha and found to be 0.81.

Data collection procedure and analysis

The Research Ethics Committee of King Fahad Hospital, Al-Ahsa, Saudi Arabia, provided ethical approval prior to data collection. For data collection, the participants were interviewed face-to-face using the Arabic-translated version of the KDQOL-36 electronic-based questionnaire via Google Forms. All in-centre haemodialysis patients who meet the inclusion and exclusion criteria and agree to participate in the study were included. For data entry, we used Excel sheets. The Statistical Package for Social Sciences (SPSS) was used to analyse the data. For continuous data, the mean and standard deviation were calculated. For categorical data, frequencies and percentages were calculated. Pearson’s Chi-square test was used to evaluate if there was a significant association between demographic characteristics and laboratory values from the QOL among patients undergoing HD. P-values of 0.05 and less were indicating a significant association.

## Results

Sixty HD participants were included in this study; of those, 43 (71.7%) were 19-59 years old and 17 (28.3%) were 60 years of age and above. Forty-one of the participants (68.3%) were male (Table 1).

**Table 1:** Demographic characteristics of the respondents in the KDQOL-36 survey (N = 60)

Demographic characteristics		n	%
Age groups	19–59 years	43	71.7
	60 years and above	17	28.3
Gender	Male	41	68.3
	Female	19	31.7

The five QOL items in the KDQOL-36 questionnaire were represented as follows: 46.7% of the patients had an average PCS while only 23.3% had a poor score. Most of the patients (61.7%) had an average score in the MCS. Meanwhile, 38.3% of the patients in BKD, 61.7% in SPKD, and 55% in EKD had good scores (Table 2).

**Table 2:** Quality of life items

Quality of life items	Score	n	%
Physical component score	Good	18	30.0
	Average	28	46.7
	Poor	14	23.3
Mental component score	Good	16	26.7
	Average	37	61.7
	Poor	7	11.7
Burden of kidney disease	Good	23	38.3
	Average	17	28.3
	Poor	20	33.3
Symptoms and problems of kidney disease	Good	37	61.7
	Average	22	36.7
	Poor	1	1.7
Effects kidney disease	Good	33	55.0
	Average	24	40.0
	Poor	3	5.0

About one-half (53.3%) of patients were on haemodialysis for two to four years and 11 months. Most of the patients (98.4%) had three sessions per week and 60% had more than three hours per session. Forty-four patients (73.3%) had residual kidney function. The dialysis access was AV fistula for 46 patients (76.7%). Most of the patients had chronic diseases (95%) such as hypertension (85%) and diabetes mellitus (56.7%). The value of the Kt/v was more than 1.2 for 47 patients (78.3%) (Table 3).

**Table 3:** Collected parameters

Collected parameters		n	%
Duration of haemodialysis	1 year and 11 months	13	21.7
	2–4 years and 11 months	32	53.3
	5 or more years	15	25.0
Number of haemodialysis per week	4 sessions	1	1.7
	3 sessions	59	98.4
Hours of haemodialysis per session	Less than 3 hours	24	40.0
	3 hours and 3:59 minutes	36	60.0
Residual kidney function	Yes	44	73.3
	No	16	26.7
Type of dialysis access	AV Fistula	46	76.7
	AV Graft	1	1.7
	Haemodialysis Catheter	13	21.7
Diabetes mellitus	No	26	43.3
	Yes	34	56.7
Hypertension	No	9	15.0
	Yes	51	85.0
Others	No	52	86.7
	Yes	8	13.3
No chronic disease	No	57	95.0
	Yes	3	5.0
Kt/v	More than 1.2	47	78.3
	Less than 1.2	13	21.7

This study revealed that patients aged 60 years and above had a good score for PCS, MCS, and EKD (p=0.023, p=0.011, and p=0.001, respectively) (Table 4).

**Table 4:** Statistically significant findings for quality-of-life measures in relation to demographic characteristics * Significant p-value < 0.05

Age groups with	in favour of		P-value
Physical component score	60 years and above	good	0.023*
Mental component score	60 years and above	good	0.011*
Effects Kidney disease	60 years and above	good	0.001*

Participants aged 60 years old and above had values of Kt/V greater than 1.2 (p=0.010). Both diabetes mellitus and hypertension were significantly correlated in favour of those aged 60 years and above (p=0.027) (Table 5).

**Table 5:** Statistically significant findings for collected parameters in relation to demographic characteristics * Significant p-value < 0.05. † Diabetes mellitus. ‡ Hypertension. ◊ Kt/V is a number used to quantify haemodialysis and peritoneal dialysis treatment adequacy.

Age groups with	in favour of		P-value
Kt/v ◊	60 years and above	more than 1.2	0.010*
Chronic Disease	60 years and above	DM† and HTN‡	0.027*

Participants who had poor scores of PCS, MCS, SPKD, and EKD had high values for haemoglobin (p=0.017, p=0.044, p=0.014, and p=0.001, respectively). Patients who had a poor score of BKD had a normal value of haemoglobin (p=0.031). Moreover, patients who had a good score for MCS, BKD, SPKD, and EKD had normal values of albumin (p=0.006, p=0.005, p=0.030, and p=0.001, respectively). In addition, patients who had a good score of EKD had a value less than 1.2 of Kt/V (p=0.001) (Table 6).

**Table 6:** Statistically significant findings for quality-of-life measures and nutritional parameters * Significant p-value < 0.05

	In favour of		P-value
Physical component score * Haemoglobin (g/dL)	Poor	High	0.017*
Mental component score * Albumin (g/L)	Good	Normal	0.006*
Mental component score * Haemoglobin (g/dL)	Poor	High	0.044*
Burden of kidney disease * Albumin (g/L)	Good	Normal	0.005*
Burden of kidney disease * Haemoglobin (g/dL)	Poor	Normal	0.031*
Symptoms and problems of kidney disease * Albumin (g/L)	Good	Normal	0.030*
Symptoms and problems of kidney disease * Haemoglobin (g/dL)	Poor	High	0.014*
Effects Kidney disease * Kt/v	Good	Less than 1.2	0.001*
Effects Kidney disease * Albumin (g/L)	Good	Normal	0.001*
Effects Kidney disease * Haemoglobin (g/dL)	Poor	High	0.001*

## Discussion

Several studies revealed that mental health, physical symptoms, and physical conditioning interact to affect health-related QOL. In this study, we noticed that nutritional parameters were found to determine impaired health-related QOL in HD patients as evaluated by scores on the KDQOL-SF-36 [25].

Many cross-sectional studies demonstrated that HD patients have lower health-related QOL outcomes than those with good nutritional status. The QOL in HD patients is associated with nutritional parameters, especially haemoglobin, albumin level and Kt/V ratio [25]. These studies utilized a variety of assessment tools to investigate the nutritional status and QOL [26,27]. Haemoglobin appears to be a contributing factor that impacts health-related QOL, which is consistent with a study conducted on four major university teaching hospitals in Australia [25]. In this study, a total of 20% of the patients had a high range of haemoglobin (> 12 g/dL) and 45% had a normal range (10-12 g/dL). High haemoglobin impacts PCS, MCS, SPKD, and EKD in favour of poor health-related QOL, but normal haemoglobin impacts BKD in favour of good QOL. A total of 33.3% of the patients had a normal range of serum albumin. This study showed that patients who had normal serum albumin levels had good scores for MCS, BKD, SPKD, and EKD. This result is compatible with other studies that correlated QOL and serum albumin in HD patients [15,16,28]. One of these studies was done by Doan et al. and revealed that there was a significant correlation between serum albumin level and QOL in 160 patients with HD. The study used the KDQOL score to measure QOL and showed that patients with hypoalbuminemia had poorer KDQOL scores [16]. Another study conducted in Gulf Cooperation Council (GCC) countries showed that low levels of albumin (<3.2 g/dL) were related to high mortality rates and low QOL [15]. However, a serum albumin level of >3.60 g/dL was associated with decreased rates of mortality [28].

In this study, the Kt/V value and EKD showed significant correlations (p=0.001). A total of 21.7% of patients had Kt/V values less than 1.2 and had good scores in EKD. A study carried out by Günalay et al. showed that there was a positive correlation between Kt/V value and QOL in HD patients (p=0.043). The study involved 50 CKD patients with HD and the European Quality of Life 5-Dimension (EQ-5D) General Quality of Life Scale was used to measure QOL [11]. Another study revealed a significant correlation between Kt/V and QOL (p=0.033) with a mean Kt/V value of 1.21 [28]. Furthermore, we observed that there was a significant association between a Kt/V of more than 1.2 and patients aged 60 years old and above (p=0.01); similarly, a study done in Brazil demonstrated a correlation between Kt/V and the age of patients (p =0.05). Younger patients had better dialysis efficacy and adequacy [29]. The diet restrictions per se in patients with ESKD could have potentially contributed to the overall poor nutritional status of these patients [30].

Chronic diseases, including hypertension and diabetes mellitus, are associated significantly with being 60 years old and above, which is consistent with a study done in GCC countries that showed that elderly patients are more prone to cardiovascular disease and diabetes mellitus [17].

One advantage of our study over previous studies is that it adds to the published data by demonstrating a significant correlation between haemoglobin, albumin, and Kt/V with each component of QOL of the KDQOL-36 questionnaire. Despite the interesting findings of this study, several limitations should be acknowledged. The retrospective-observational nature of this study precludes causal inference of associated relationships between nutritional parameters and health-related QOL. Also, the relatively small sample size in a single centre (N = 60) and the possibility of confounding bias are factors that might limit the generalizability and strength of these findings.

## Conclusions

CKD can lead to major complications including ESKD which can be treated by HD. A significant reduction of nutritional parameters can be developed from HD, which can contribute to poor health-related QOL. Our study shows each element of QOL by using the KDQOL-36 questionnaire in HD patients and its significant correlation with haemoglobin, albumin, and Kt/V. This emphasizes the significance of nutritional status in HD patients and provides a better understanding of factors affecting health-related QOL in Al-Ahsa.
